# Dissecting Gene Expression Changes Accompanying a Ploidy-Based Phenotypic Switch

**DOI:** 10.1534/g3.116.036160

**Published:** 2016-11-11

**Authors:** Gareth A. Cromie, Zhihao Tan, Michelle Hays, Eric W. Jeffery, Aimée M. Dudley

**Affiliations:** * Pacific Northwest Research Institute, Seattle, Washington 98122; † Institute of Medical Biology, Agency for Science, Technology and Research, Singapore; ‡ Molecular and Cellular Biology Program, University of Washington, Seattle, Washington

**Keywords:** aneuploidy, gene expression, colony morphology, RNA-seq

## Abstract

Aneuploidy, a state in which the chromosome number deviates from a multiple of the haploid count, significantly impacts human health. The phenotypic consequences of aneuploidy are believed to arise from gene expression changes associated with the altered copy number of genes on the aneuploid chromosomes. To dissect the mechanisms underlying altered gene expression in aneuploids, we used RNA-seq to measure transcript abundance in colonies of the haploid *Saccharomyces cerevisiae* strain F45 and two aneuploid derivatives harboring disomies of chromosomes XV and XVI. F45 colonies display complex “fluffy” morphologies, while the disomic colonies are smooth, resembling laboratory strains. Our two disomes displayed similar transcriptional profiles, a phenomenon not driven by their shared smooth colony morphology nor simply by their karyotype. Surprisingly, the environmental stress response (ESR) was induced in F45, relative to the two disomes. We also identified genes whose expression reflected a nonlinear interaction between the copy number of a transcriptional regulatory gene on chromosome XVI, *DIG1*, and the copy number of other chromosome XVI genes. *DIG1* and the remaining chromosome XVI genes also demonstrated distinct contributions to the effect of the chromosome XVI disome on ESR gene expression. Expression changes in aneuploids appear to reflect a mixture of effects shared between different aneuploidies and effects unique to perturbing the copy number of particular chromosomes, including nonlinear copy number interactions between genes. The balance between these two phenomena is likely to be genotype- and environment-specific.

Chromosome missegregation during meiosis or mitosis can produce cells that are aneuploid, containing a chromosome complement that is not a multiple of the haploid count. This often results in strong phenotypic effects. In humans, inheritance of a single copy of any autosome (monosomy) or three copies of most autosomes (trisomy) is an embryonic lethal event. The most common, viable, inherited aneuploidy is trisomy 21, which leads to the collection of phenotypes that comprise Down syndrome. In addition, several imbalances in sex chromosomes are viable, including Turner Syndrome (X0) and Klinefelter Syndrome (XXY).

While meiotic chromosome missegregation events lead to inherited aneuploidy, mitotic missegregation produces aneuploid subpopulations of cells and mosaicism. This phenomenon is strongly associated with cancer. Tumors show a very high frequency of aneuploidy, but the degree to which this aneuploidy is simply a result of the disordered cellular processes in cancer *vs.* actually promoting cellular transformation is unclear (Gordon *et al.* 2012).

The mechanism linking aneuploidy to phenotype is believed to operate primarily through changes in gene expression. By altering the copy number of a subset of genes, relative to the rest of the genome, aneuploidy causes a change in the relative expression level of those genes. In the case of Down syndrome, for example, the relative expression of genes on chromosome 21 has been shown to be increased (Mao *et al.* 2003), mirroring the increased copy number of those genes. This increase in gene expression can produce phenotypic effects in several ways. One mechanism is via direct downstream effects of the overexpressed genes. For example, individuals with Down syndrome show increased risk for early onset dementia, most likely because the gene encoding the amyloid precursor protein is present on chromosome 21 and therefore has elevated expression in trisomic individuals (Salehi *et al.* 2006). In addition, work in model organisms has shown that increasing the expression level of regulatory genes, such as transcription factors, can lead to a cascade of downstream effects across the rest of the genome, also producing phenotypic effects (Rancati *et al.* 2008). However, for most of the phenotypes associated with Down syndrome, or other aneuploidies, neither the identity of the relevant overexpressed genes, nor the mechanism by which their overexpression produces the phenotype, are understood.

Dissecting the mechanisms through which aneuploidy exerts phenotypic effects in humans is limited by technical and ethical constraints. However, work in model organisms, particularly yeast, has provided several insights into these questions. These studies have revealed that many of the phenotypes associated with the transition from euploidy to aneuploidy are specific to particular aneuploid states (Pavelka *et al.* 2010). However, it also appears that some phenotypic effects are common to multiple aneuploid states. In general, aneuploid cells appear to suffer proliferation and growth defects, although the intensity of these effects varies across specific aneuploid states and genotypes (Torres *et al.* 2007; Pavelka *et al.* 2010; Sheltzer *et al.* 2012; Hose *et al.* 2015). Similarly, many aneuploid cells appear to suffer from protein transport defects (Dodgson *et al.* 2016) and proteotoxic stress, with the latter perhaps caused by imbalances in protein stoichiometry in multi-subunit complexes (Sheltzer and Amon 2011; Oromendia *et al.* 2012).

In any given environment, the fitness of an aneuploid appears to reflect a combination of these general negative impacts of aneuploidy along with any phenotypes associated with the particular pattern of chromosome (and hence gene) copy number change. With sufficiently strong selective pressure, the benefits of the specific effects can sometimes outweigh the general costs of aneuploidy, giving aneuploids a selective advantage (Pavelka *et al.* 2010; Sheltzer and Amon 2011). If selection is then removed, a return to euploidy will be favored, while if selection is maintained, the cells appear to respond either by finding an alternative, nonaneuploid solution to the selective pressure (Yona *et al.* 2012), or by maintaining aneuploidy but ameliorating the associated negative effects (Torres *et al.* 2010).

In a previous study, we identified a budding yeast system in which transition between euploid and aneuploid states is associated with a strong phenotypic response: a dramatic change in colony morphology (Tan *et al.* 2013). Our original, euploid strain produces colonies with a complex “fluffy” morphology, while certain disomic derivatives of this strain display the “smooth” colony morphology characteristic of most laboratory strains. We were able to demonstrate that, while disomy of several different chromosomes induced this phenotypic switch, disomy of several others did not. This argues that the phenotypic switch is not the result of some general effect of the aneuploid state, but rather a response to changes in the expression of dosage-sensitive genes that affect colony morphology. In support of this gene-dosage model, we found that the effect on colony morphology caused by gaining an extra copy of chromosome XVI could be replicated by increasing the copy number of a single gene on that chromosome, *DIG1*, a transcriptional regulator of colony morphology, in an otherwise haploid (euploid) genome. However, because deleting one copy of *DIG1* in a chromosome XVI disome only partially restored the fluffy phenotype of F45, we hypothesized that other dosage-sensitive genes on that chromosome also affect colony morphology (Tan *et al.* 2013).

Here, we use RNA-seq to characterize and dissect the specific factors underlying the transcriptional effects of gaining an extra copy of two different chromosomes (XV and XVI) in the F45 genetic background. Each of these disomies causes F45 to adopt a smooth colony morphology. We expected that some of the factors driving differences in gene expression between the euploid and disomic colonies would be shared between the two disomes. These include nonspecific stress responses to aneuploidy and shared environmental effects operating in the smooth (disomic) *vs.* fluffy (euploid) colonies. We expected that other factors would be specific to each disome, including direct copy number effects affecting genes on the disomic chromosomes themselves and downstream effects of altering the copy number of regulatory genes on the disomic chromosomes. We also specifically investigated how copy number changes affecting a transcriptional regulatory gene (*DIG1*) interact with the copy number of other genes on the same chromosome.

## Materials and Methods

### Yeast strains and media

Unless noted, standard media and methods were used for growth and genetic manipulation of yeast (Rose 1990). The strains used in this study are listed in Table 1.

**Table 1:** Yeast strains used in this study

Strain Name	Progenitor	Genotype	Karyotype	Source
YPG725 (F45)		*MAT***a** *ho*∆*0*::*hphMX6*, *SPS2:EGFP:natMX4*, unmapped serine auxotrophy	Euploid haploid	Tan *et al.* (2013)
YO1853	F45	*MAT***a** *ho*∆*0*::*hphMX6*, *SPS2:EGFP:natMX4*, unmapped serine auxotrophy, [pRS41K]	Euploid haploid, plasmid pRS41K	Taxis and Knop (2006) plasmid only
YO1773	F45	*MAT***a** *ho*∆*0*::*hphMX6*, *SPS2:EGFP:natMX4*, unmapped serine auxotrophy, [DIG1-pFA6a-KanMX4]	Euploid haploid, plasmid pAB340	Tan *et al.* (2013)
YO902	F45	*MAT***a** *ho*∆*0*::*hphMX6*, *SPS2:EGFP:natMX4*, unmapped serine auxotrophy	Disomy XV	Tan *et al.* (2013)
YO785	F45	*MAT***a** *ho*∆*0*::*hphMX6*, *SPS2:EGFP:natMX4*, unmapped serine auxotrophy	Disomy XVI	Tan *et al.* (2013)
YO1000	F45	*MAT***a** *ho*∆*0*::*hphMX6*, *SPS2:EGFP:natMX4*, unmapped serine auxotrophy, *dig1Δ*::*kanMX4/DIG1*	Disomy XVI	Tan *et al.* (2013)
YO2085	F45	*MAT***a** *ho*∆*0*::*hphMX6*, *SPS2:EGFP:natMX4*, unmapped serine auxotrophy, *flo11Δ0*::*kanMX4*	Euploid haploid	This study
YO2086	F45	*MAT***a** *ho*∆*0*::*hphMX6*, *SPS2:EGFP:natMX4*, unmapped serine auxotrophy, *flo11Δ0*::*kanMX4*	Euploid haploid	This study
YO2088	F45	*MAT***a** *ho*∆*0*::*hphMX6*, *SPS2:EGFP:natMX4*, unmapped serine auxotrophy, *cis3*Δ*0*::*kanMX4*	Euploid haploid	This study
YO2089	F45	*MAT***a** *ho*∆*0*::*hphMX6*, *SPS2:EGFP:natMX4*, unmapped serine auxotrophy, *cis3*Δ*0*::*kanMX4*	Euploid haploid	This study

### RNA preparation and sequencing

After 4 d of growth on YPD (2% glucose) plates at 30°, whole colonies were harvested by scraping the surface of the agar plate. To obtain sufficient amounts of RNA, 3–5 colonies were pooled for each sample. Samples were collected and processed in the following batches, each containing euploid, wild-type F45 as a control. The first batch consisted of four replicates of: YPG725 (F45), YO902 (disome XV), YO785 (disome XVI), YO1000 (disome XVI, hemizygous *dig1*Δ), YO1853 (control plasmid), and YO1773 (*DIG1* plasmid) (Tan *et al.* 2013). The second batch consisted of three replicates of YPG725 (F45), and two replicates of YO2085 (*flo11*Δ), YO2086 (*flo11*Δ), YO2088 (*cis3*Δ), and YO2089 (*cis3*Δ). Total RNA was prepared as described previously (Swanson *et al.* 1991).

Following extraction, total RNA from the pooled colonies was quantified by Bioanalyzer (Agilent). Total RNA for each sample (5 µg) was then processed using the Tru-Seq stranded mRNA kit (Illumina) following the manufacturer’s instructions. Individual sample libraries were pooled for sequencing. The first batch was analyzed by paired-end, 51 nucleotide read sequencing in one lane of an Illumina HiSeq2000. The second batch was analyzed by paired-end, 75 nucleotide read sequencing on an Illumina NextSeq 500.

### Extending the reference genome and annotation

Before read-pair alignment, the S288c GFF (General Feature Format) file was extended to cover additional classes of noncoding RNA, specifically CUTs and SUTs (Xu *et al.* 2009), MUTs (Lardenois *et al.* 2011), and XUTs (van Dijk *et al.* 2011), using data downloaded from the *Saccharomyces* Genome Database (SGD) on 4/28/2014. Additionally, the reference genome fasta and GFF files were extended to include genes present in F45, but absent in S288c. Note that the chromosomes encoding these genes are unknown. We carried out whole-genome sequencing of F45 using a MiSeq (Illumina) with a fragment size range of ∼600–800 bp and paired-end 250 bp reads. Reads were trimmed for adaptor sequences using cutadapt (v. 1.7.1) (Marcel 2011) and aligned to the S288c reference using the BWA (v. 0.7.5a) (Li and Durbin 2009) mem command. Read-pairs where neither read aligned to the reference genome then underwent *de novo* assembly using IDBA (v. 1.1.1) (Peng 2010) with the parameters–mink 23–maxk 35–step 2–seed_kmer 23. Nucleotide BLAST was used to identify potential ORFs in the novel contigs, and these ORFs were appended to both the reference sequence and the annotation file (Supplemental Material, File S1 and File S2). The aligned reads are available from the European Nucleotide Archive (ENA) under study PRJEB15176.

### Read-pair alignment

Read-pair alignments for RNA-seq data were carried out against the S288c reference (R64-1-1), extended as described above, using Bowtie2 (version 2.1.0) (Langmead and Salzberg 2012) with the parameters [-N 1 -I 50 -X 450 -p 6–reorder -x -S] and allowing one mismatch per read. For each strain, read alignments were converted to gene counts using featureCounts (version 1.4.0) in the Subread package (Liao *et al.* 2014), with the parameters [-a -o -t gene –g ID –s 2 -T 1 -p -P -d 50 -D 450]. Reads were not filtered based on mapping quality, and we are thus cautious in our interpretation of counts of genes that have paralogs with similar sequences, or which contain large low complexity regions. Read sequences and gene count tables are available from the Gene Expression Omnibus (GEO) under accession GSE85843.

### Differential expression analysis

Analysis of differential gene expression was carried out using edgeR [v. 3.6.8] (Robinson *et al.* 2010) based on the tables of raw counts produced by featureCounts (*Materials and Methods*). Separate data normalization and analyses were conducted for the first and second sample batches, each of which included a wild-type control (F45 or F45 with empty vector). Library sizes were normalized using calcNormFactors, with only nuclear-encoded ORFs (genes with systematic names beginning with “Y”) used to estimate the normalization factor and excluding chromosomes XV and XVI for the first batch of samples, which included the disomic strains. This normalization factor was then applied to all genes and dispersion parameters were estimated using the estimateGLMTrendedDisp and estimateGLMTagwiseDisp commands. To identify genes differentially expressed between strains, we conducted pairwise testing using the glmFit and glmLRT commands, with a p-value cutoff of 0.01 (after Benjamini–Hochberg multiple hypothesis correction, *i.e.*, false discovery rate). Because principal coordinate analysis using moderated (plus two counts per gene) counts per gene per million reads demonstrated strong clustering on the first principal component (PC) of strains YO285 and YO286 (*flo11*Δ) *vs.* F45, and strains YO2088 and YO2089 (*cis3*Δ) *vs.* F45, RNA-seq data from each of these pairs were combined for further analysis. When plotting log_2_ fold changes on scatterplots, calculating regression parameters, correlation coefficients, Wilcoxon rank sum tests, medians, and SD, only nuclear-encoded ORFs, present in the reference genome with median basal expression of at least one read per million reads in F45 (first batch), were included. In addition, for each genotype, the variance of the log_2_ moderated (plus two counts per gene) counts per gene per million reads was calculated for each gene, and genes with variances ≥ 3 were removed from any analysis using data from that genotype.

### Differential expression from disomic chromosomes

For chromosomes I–XIV, genes differentially expressed at the p < 0.01 level with a minimum expression change of twofold between the two disomes, or between the disomes and F45, were identified as above. To identify differential expression of genes on chromosomes XV and XVI, independent of the direct effect of copy number, normalization was repeated for each disome using twice the value calculated by calcNormFactors applied to chromosomes I–XIV. These normalized values were used for comparisons involving the disomic chromosomes. The genes identified in this way as differentially expressed from chromosomes XV and XVI were added to the list from chromosomes I–XIV.

### Functional enrichment of gene lists

We looked for functional enrichment of *Saccharomyces cerevisiae* gene lists using g:Profiler (http://biit.cs.ut.ee/gprofiler/). Holm–Bonferroni corrected Enrichment p–values < 0.05 were accepted, with moderate hierarchical filtering.

### Principal component analysis (PCA)

PCA was carried out using the princomp command in R (version 3.1.1, (R Core Team 2014). Loadings for the hemizygous *DIG1/dig1*Δ strain, chromosome XVI disome, *DIG1*-overexpressing strain, and the empty plasmid control were calculated using only nuclear encoded-ORFs with median basal expression of at least one read per million reads in F45 (first batch). These loadings were then applied to the whole dataset. Significant nonzero values on the second *DIG1* PC were identified using the generalized linear model functionality of edgeR (Robinson *et al.* 2010). Normalization for library size (calcNormFactors) was carried out using only nuclear-encoded ORFs (genes with systematic names beginning with “Y”) and excluding chromosome XVI. This normalization was then applied to all genes. Dispersion parameters were estimated using estimateGLMTrendedDisp and estimateGLMTagwiseDisp commands. Significant nonzero values on the second *DIG1* PC were identified using the glmLRT command with a p-value cutoff of 0.01 (after Benjamini–Hochberg multiple hypothesis correction). Treating the intercept as zero, from the regression line corresponding to the first PC we expect that 1 × (hemizygous *DIG1/dig1*Δ strain − chromosome XVI disome expression) + 0.54 × (*DIG1*-overexpressing − empty plasmid control expression) will equal zero when a zero value occurs on the second PC. Therefore, significant nonzero values in the second PC were identified using glmLRT with the hemizygous *DIG1/dig1*Δ strain, the chromosome XVI disome, the *DIG1*-overexpressing strain, and the empty plasmid control strain given the weights 1, −1, 0.54, and −0.54 to reflect the regression relationship.

### Data availability

The datasets generated during and/or analyzed in the current study are available in the GEO repository under GSE85843 and ENA repository under PRJEB15176.

## Results

**Figure 1:**
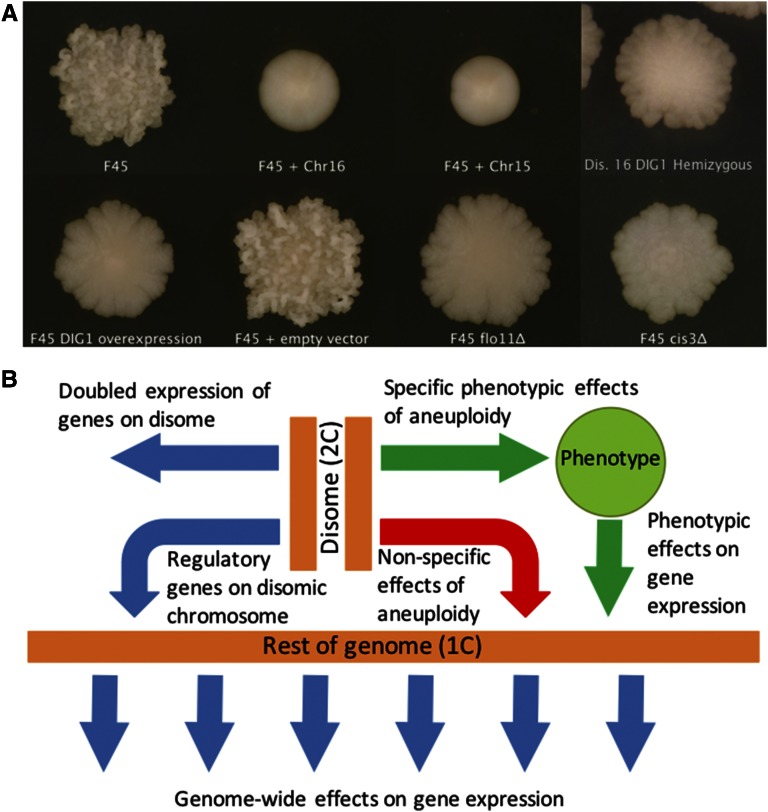
(A) Strain colony morphologies. All images were taken with a Canon PowerShot SX101S. (B) Model showing different mechanisms by which aneuploidy can affect gene expression. Chr, chromosome.

We previously isolated several disomic derivatives of the strain F45, including one harboring an extra copy of chromosome XV and another harboring an extra copy of chromosome XVI (Tan *et al.* 2013). Because these disomies arose by mitotic missegregation, the two copies of the disomic chromosomes are homozygous. Euploid F45 produces colonies with a strong “fluffy” phenotype, characterized by an intricate pattern of ruffles and channels. In contrast, the chromosome XV and XVI disomes both produce smooth, unstructured colonies similar to commonly used laboratory strains (Figure 1A). To characterize gene expression differences between these strains, we performed RNA-seq analysis on RNA isolated from fully developed colonies grown on solid medium (YPD, 2% glucose) for 4 d. Because F45 shows significant sequence divergence from the *S. cerevisiae* reference genome, we also performed whole-genome sequence analysis and *de novo* assembly of the F45 strain background. This analysis predicted several genes not present in the S288c genome, and these nonreference genes were added to our expression analysis pipeline (*Materials and Methods*).

In principle, the transcriptional profile of any given disome could be composed of several distinct factors, with some unique to each karyotype and others shared by multiple aneuploid states (Figure 1B). First, there are *cis* effects on the expression of genes on the disomic chromosomes themselves, which should be increased ∼twofold compared to an isogenic euploid strain. Second, there are *trans* effects caused by the differential expression of regulatory genes on the disomic chromosomes, which could produce a large number of downstream effects on transcription across the whole genome. Both of these effects would be expected to be largely disome-specific. Third, either of the first two mechanisms could produce phenotypic effects that, in turn, could feed-back on the expression of additional genes. In such a case, different disomes that place cells in the same phenotypic state might be expected to share an associated transcriptional response. Fourth, general transcriptional responses to the aneuploid state, which are not specific to changing the copy number of specific chromosomes, have been described (Torres *et al.* 2007) (Hose *et al.* 2015). Responses of this type should be shared by many, if not all, disomes.

### Gene expression from disomic chromosomes is elevated twofold

**Figure 2:**
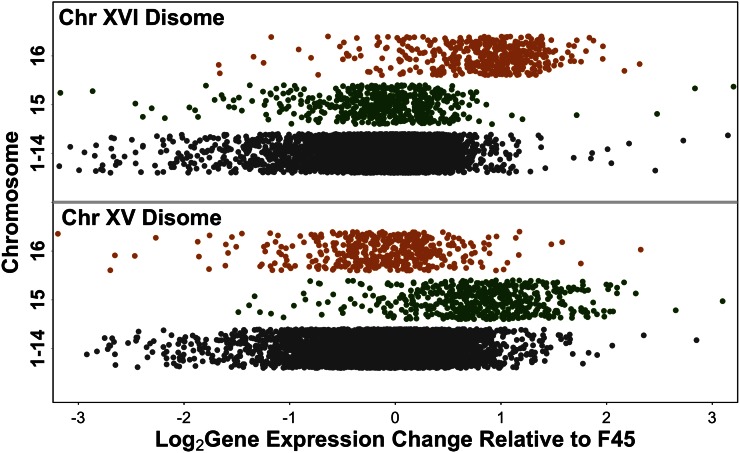
Gene expression from the disomic chromosomes is ∼twofold higher than from the rest of the genome. Chromosome XV expression is shown in green, chromosome XVI expression in orange, and expression from the other chromosomes in gray.

Consistent with other studies (Hughes *et al.* 2000; Torres *et al.* 2007, 2016; Rancati *et al.* 2008; Dephoure *et al.* 2014), the average expression of genes on the disomic chromosomes was increased relative to the F45 euploid (median 1.79-fold for disome XV and median 1.86-fold for disome XVI) (Figure 2). To control for shared effects on expression, such as those that might be caused by the smooth colony morphology of the disomes or shared effects of the aneuploid state, we then compared gene expression on chromosomes XV and XVI between the two disomes themselves. Genes on chromosome XV had a median expression level 1.98-fold higher in the strain disomic for chromosome XV, and genes on chromosome XVI had a median expression level 1.96-fold higher in the chromosome XVI disome. The SD of the F45:disome log_2_ ratios (0.65 for chromosome XV and 0.59 for chromosome XVI) was decreased (0.55 and 0.52, respectively) in the disome:disome comparisons. Therefore, as expected, the effect of doubling chromosome copy number, when isolated from other factors, is to double gene expression levels from the affected chromosome. However, the difference between the SD of the disome:F45 comparisons and the disome:disome comparisons suggests that factors shared between the two disomes do contribute significantly to the differential expression of genes on chromosomes XV and XVI, relative to F45, independent of the copy number of those genes.

### Role of copy number changes of the DIG1 transcriptional repressor gene in the disome XVI transcriptional profile

*DIG1*, which encodes a transcriptional repressor of the “filamentous growth” MAPK pathway (Cook *et al.* 1996; Granek and Magwene 2010; Tan *et al.* 2013), is known to regulate complex colony morphology (Granek and Magwene 2010; Tan *et al.* 2013) and is located on chromosome XVI. We previously demonstrated that overexpression of *DIG1*, in an F45 euploid background, largely recapitulates the phenotypic effect of the full chromosome XVI disome, *i.e.*, produces smooth colonies (Tan *et al.* 2013). Similarly, deletion of a single copy of *DIG1* in a chromosome XVI disome partially restores the fluffy phenotype of F45, producing colonies with fluffy perimeters, but smooth centers (Tan *et al.* 2013) (Figure 1A).

**Figure 3:**
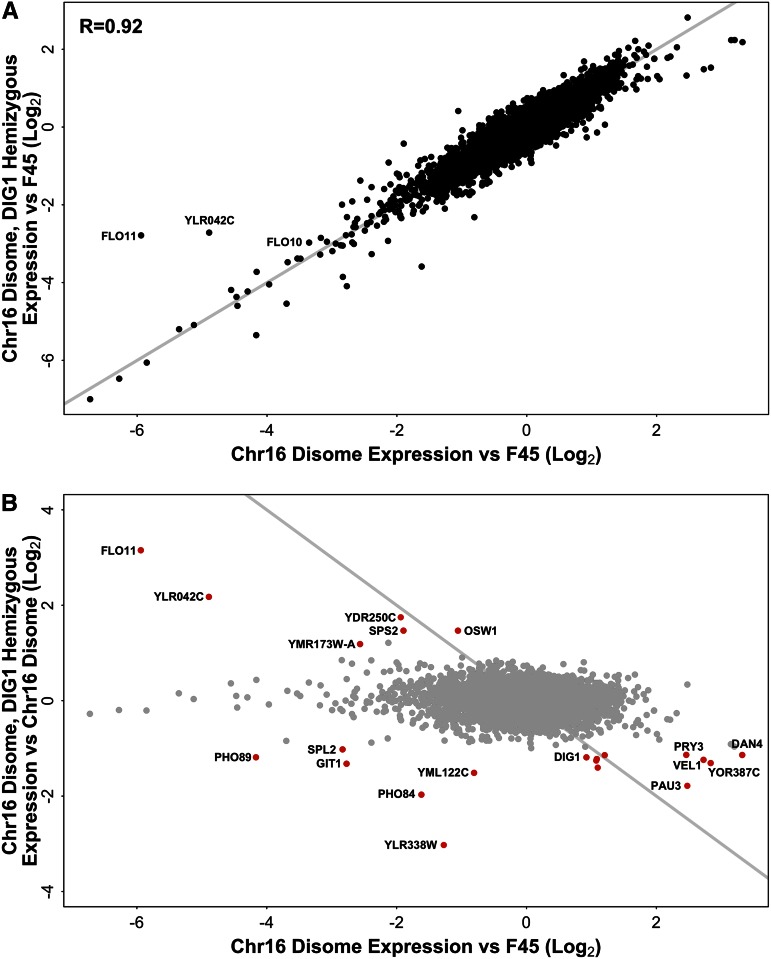
(A) Differential gene expression in the chromosome XVI disome and the hemizygous *DIG1*/*dig1*Δ derivative of the chromosome XVI disome, both relative to F45. Genes on disomic chromosome XVI had expression ratios adjusted downward twofold. *x* = *y* line shown in gray. (B) Differential gene expression in the chromosome XVI disome, relative to F45 and the hemizygous *DIG1* deletion, relative to the full chromosome XVI disome. Genes on disomic chromosome XVI had expression ratios adjusted downward twofold in the disome *vs.* F45 dataset. Red points are genes whose expression changed significantly, and >twofold (up or down), between the chromosome XVI disome and the disome with the hemizygous *DIG1* deletion. Gray line indicates F45 expression level. Chr, chromosome.

One of the mechanisms that we expected to drive disome-specific gene expression changes was through copy number changes of regulatory genes found on the disomic chromosome (Figure 1B). To investigate the contribution of changes in the copy number of a chromosome XVI regulatory gene to the differential transcription profile of the full chromosome XVI disome, we used RNA-seq to analyze the expression profile of colonies grown on solid agar in a chromosome XVI disome that was hemizygous for *DIG1* (*DIG1/dig1*Δ). As expected, expression of *DIG1* itself was increased ∼twofold (1.90-fold) in the disome, relative to F45, and then reduced ∼twofold (2.28-fold) in the hemizygous deletion (Figure 3B). Relative to euploid F45, the differential expression profile of the hemizygous *DIG1* chromosome XVI disome was extremely similar to that of the chromosome XVI disome, which harbored both copies of *DIG1* (*R* = 0.92, Pearson test, 2-tailed, *t* = 174.3026, df = 5777, p-value < 1e−15) (Figure 3A). Therefore, despite the pronounced effect of *DIG1* copy number on the colony morphology, the transcriptional differences between F45 and the chromosome XVI disome are largely independent of the effect of the copy number of *DIG1*.

Closer examination of the data, however, indicated that, among the genes most strongly induced in the full chromosome XVI disome, relative to F45, most did show a reduction in expression in the *DIG1* hemizygous strain (Figure 3, A and B). The genes in this group (*ZPS1*, *YOR387C*, *VEL1*, *DAN1*, *DAN4*, *PAU3*, *PRY3*, *YFL051C*, and *YNL150W*) mostly encode cell surface proteins, with expression of the first three responding strongly to zinc levels (Lyons *et al.* 2000). However, consistent with the incomplete restoration of the fluffy phenotype in the hemizygous strain, gene expression levels were generally not fully restored to the F45 euploid level (Figure 3B, bottom right).

In contrast to the effect of *DIG1* hemizygosity on genes induced in the chromosome XVI disome, its effect on expression of genes strongly repressed in the disome was more complex. Most genes strongly repressed in the chromosome XVI disome, relative to F45, showed a similarly low level of expression in the hemizygous strain (Figure 3, A and B). However, a small number of repressed genes were significantly (p < 0.01, after multiple hypothesis correction) and substantially (>twofold) more highly expressed in the hemizygous strain. This set of genes (Figure 3B) included *FLO11*, which encodes a flocculin that plays a critical role in complex colony morphology (Lo and Dranginis 1998; Granek and Magwene 2010; Voordeckers *et al.* 2012), the *SPS2* and *OSW1* genes, which are required for the construction of spore walls (Coluccio *et al.* 2004), *YLR042C*, which encodes an uncharacterized cell wall protein, and two dubious ORFs (*YDR250C* and *YMR173W-A*). Complex colony formation is known to be dependent on cell wall genes such as the flocculins (Guo *et al.* 2000; Voordeckers *et al.* 2012), and it seems likely that among the cell wall genes identified in this group are genes important for the partial restoration of the fluffy phenotype seen in the hemizygous *DIG1* deletion. As with the set of genes that showed reduction in the *DIG 1* hemizygous strain, expression of these genes was changed relative to the full disome, but not fully restored to their F45 euploid levels (Figure 3B). Interestingly, expression of the *FL010* flocculin, which was strongly repressed in the chromosome XVI disome relative to F45, was essentially unaffected by the *DIG1* hemizygous deletion, remaining just as repressed relative to F45 (Figure 3A).

A final group of genes were both strongly repressed by the chromosome XVI disome, relative to F45, and substantially (>twofold) and significantly (p < 0.01, after multiple hypothesis correction) more strongly repressed in the hemizygous *DIG1* strain, relative to the disome harboring two copies of *DIG1* (Figure 3B, bottom left). This group consisted of *PHO84*, *PHO89*, *SPL2*, and *GIT1*, genes involved in phosphate transport that are induced in response to low phosphate conditions (Almaguer *et al.* 2003; Ljungdahl and Daignan-Fornier 2012), and two dubious ORFs (*YLR338W* and *YML122C*). The expression of these genes appears to respond to a nonlinear interaction between *DIG1* copy number and that of one or more other genes on chromosome XVI (see below).

### Comparing the effect of changing DIG1 copy number in euploid F45 *vs.* in the chromosome XVI disome

Our previous study found that reducing the copy number of *DIG1*, via a hemizygous deletion in the context of a chromosome XVI disome, partially converted the smooth morphology of the disome back to the fluffy phenotype of F45 (Tan *et al.* 2013). However, moderate (∼two- to fivefold) overexpression of *DIG 1* in euploid F45, from a low copy number plasmid, caused a stronger change in colony morphology from fluffy to fully smooth (Tan *et al.* 2013) (Figure 1A). To compare the effect of perturbing the copy number of *DIG1* in the euploid *vs.* the chromosome XVI disome genetic backgrounds, we carried out RNA-seq and differential expression analysis on smooth colonies from the *DIG1* overexpression strain.

**Figure 4:**
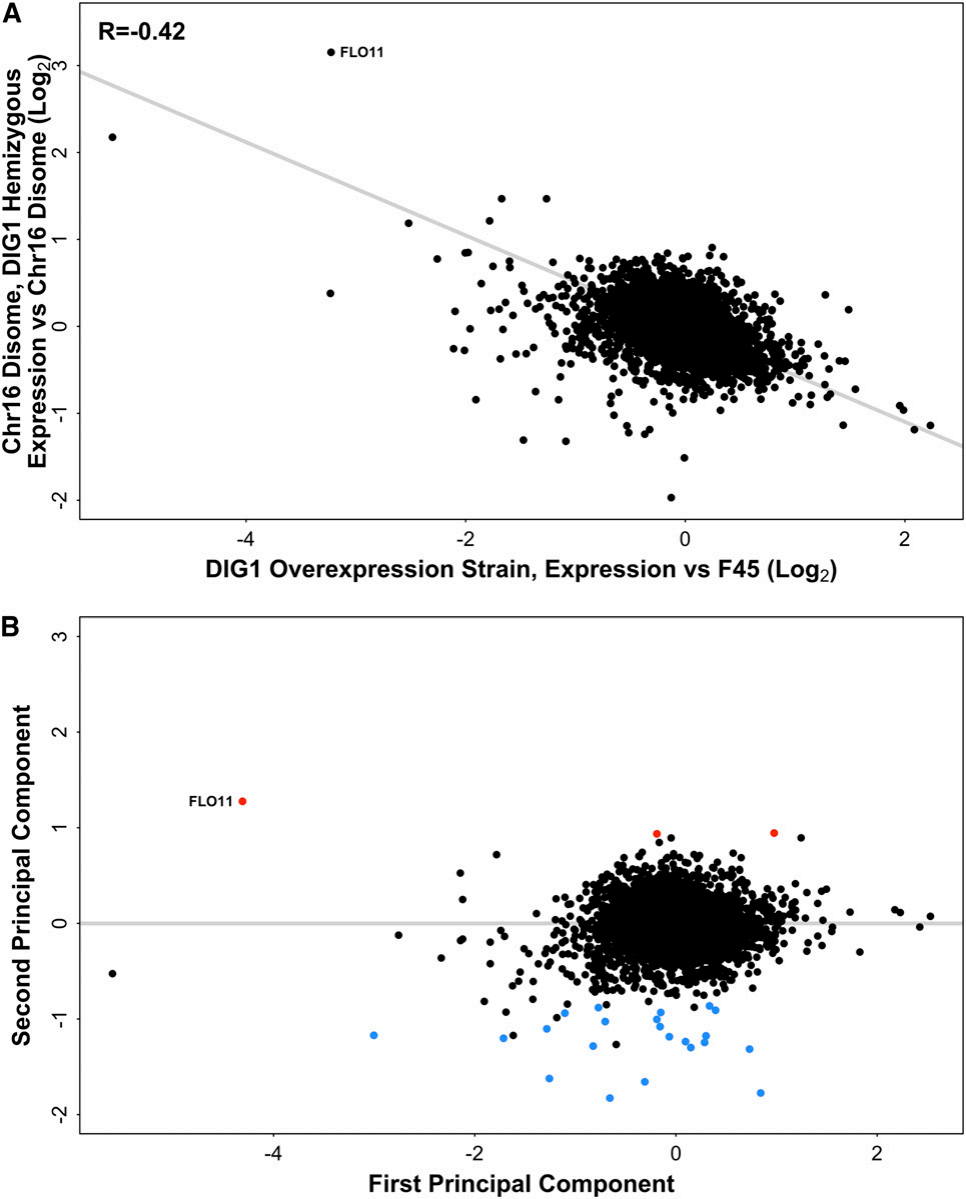
(A) Differential gene expression in the *DIG1* overexpression strain, relative to F45 (with empty vector) and the hemizygous *DIG1* derivative (*DIG1/dig1*Δ) of the chromosome XVI disome, relative to the full chromosome XVI disome. Gray line indicates TLS regression. (B) Rotation of the data using PCA. First principal component (*x*-axis) is equivalent to the TLS regression line in (A). Genes with significantly higher than expected expression in the hemizygous *DIG1* strain are shown in red, genes with significantly lower than expected expression in blue. PCA, principal component analysis; TLS, total least squares.

Comparing the effects of *DIG1* overexpression, relative to euploid F45, to the effect of the hemizygous *DIG1* deletion, relative to the full chromosome XVI disome (Figure 4A), revealed a modest but significant correlation between the effects of the two perturbations (*R* = −0.42, Pearson test, two-tailed, *t* = −34.917, df = 5777, p-value < 1e−15). As expected from the opposite directions of effect on *DIG1* copy number (overexpression *vs.* copy number reduction), this correlation was negative. However, the weakness of this correlation suggests that the effect of perturbing *DIG1* copy number might depend on the copy number of the genes on the rest of chromosome XVI.

Dig1 binds the Ste12 transcription factor and represses transcriptional activation of Ste12 target genes, including targets of the Ste12-Tec1 complex with roles in complex colony morphology (Madhani and Fink 1997; Chou *et al.* 2006). Examining 41 genes with conserved Ste12 and Tec1 binding motifs (Chou *et al.* 2006), we observed a significant overrepresentation (29/2197 *vs.* 12/4388; Fisher’s Exact Test, two-tailed, p-value < 1.1e−06) of these genes among the set of genes, including *FLO11* and *YLR042C* (Figure 4A), which were both repressed in the *DIG1* overexpression strain (log_2_FC < 0) and induced in the hemizygous *DIG1*/*dig1*Δ strain (log_2_FC >0), relative to F45 and the chromosome XVI disome harboring both copies of *DIG1*, respectively.

The effect on transcription of the *DIG1* overexpression strain appears to be stronger than the effect of the *dig1* hemizygous deletion (Figure 4A), as total least squares (TLS) regression had a slope of −0.54. This is consistent with the larger change in *DIG1* expression level in the overexpression strain (4.25-fold up, relative to F45) compared with the hemizygous deletion (2.28-fold down, relative to the full chromosome XVI disome). A simple model for *DIG1* action would suggest that plotting the (log) effects of halving *DIG1* expression against the effects of doubling *DIG1* expression would give a regression slope of −1, *i.e.*, reflecting the ratio of the log changes in *DIG1* expression. Given the *DIG1* expression changes observed in the hemizygous deletion and overexpression strains, this model predicted a slope of −0.57, and an intercept of 0, for the regression in Figure 4A, very close to the observed values of −0.54 and −0.025. Interestingly, however, there were several strong outliers from the regression trend. These included *FLO11*, whose expression level was changed by almost exactly the same amount after both perturbations, rather than showing a larger relative transcriptional change in the *DIG1* overexpression strain, as expected from the TLS regression slope (Figure 4A).

### Gene expression changes reflecting a nonlinear interaction between chromosome XVI ploidy and DIG1 expression level

To further characterize the differences between the effects of perturbing *DIG1* copy number in the disome *vs.* euploid F45, we performed a principle component analysis (PCA) that was mathematically equivalent to rotating the data in Figure 4A to make the TLS regression line into the new *x*-axis (Figure 4B) (*Materials and Methods*). The first PC (Figure 4B, *x*-axis) then captures the major axis of covariation between the effect of the *DIG1* hemizygous deletion and the effect of *DIG1* overexpression. The second PC (Figure 4B, *y*-axis) captures variation orthogonal to this first axis, *i.e.*, behavior different to that expected from the major trend. Genes whose expression responds nonlinearly to the two perturbations of *DIG1* copy number (in the two different genetic backgrounds) should score highly on this second PC. Because the magnitudes of the two perturbations of *DIG1* expression level are different (4.25-fold *vs.* 2.28-fold), one explanation for the behavior of these genes could be that they are regulated solely by *DIG1* expression level, but that the relationship between their expression and that of *DIG1* is nonlinear. Alternatively, the behavior of these genes could represent a nonlinear interaction between *DIG1* expression level and the expression level of other regulatory genes on chromosome XVI.

To identify genes with values significantly different to zero in the second PC, we used the generalized linear model functionality of EdgeR (Robinson *et al.* 2010) with the observed regression slope of −0.54 and treating the intercept as zero (*Materials and Methods*). These genes can then be split into two groups, those showing an expression change in the hemizygous *DIG1* deletion that is either higher or lower than expected, based on their behavior in the *DIG1* overexpression strain. In the first group, we observed only three genes whose expression change was more than twofold higher than expected in the hemizygous *DIG1* deletion, based on their behavior in the *DIG1* overexpression strain. This group consisted of *FLO11* and the ammonium permease *MEP2*, both known to have roles in complex colony morphology (Lo and Dranginis 1998; Lorenz and Heitman 1998a; Voordeckers *et al.* 2012), and the poorly characterized gene *SPG1* (Figure 4, A and B). In the second group, we observed 20 genes (plus three dubious ORFs) whose expression change was more than twofold lower than expected in the hemizygous *DIG1* deletion, based on their behavior in the *DIG1* overexpression strain. This set included *FLO10*, which was strongly repressed by overexpressing *DIG1*, but whose expression was relatively unaffected by the hemizygous *DIG1* deletion, relative to the full disome. The remaining 19 genes in this group were all repressed, to a greater or lesser degree, both by the hemizygous *DIG1* deletion (relative to the full chromosome XVI disome) and also by *DIG1* overexpression (relative to F45), rather than showing the expected opposite directions of effect (Figure 4, A and B).

The 19 genes repressed both by the hemizygous *DIG1* deletion and by *DIG1* overexpression included the phosphate transport genes (*PHO84*, *PHO89*, *SPL2*, and *GIT1*) identified earlier (Figure 3B), along with another phosphate gene, *PHO5*, and *VTC1*, encoding a vacuolar transport protein involved in vacuolar polyphosphate accumulation. Similarly, another subset of these genes are induced by low zinc availability (Lyons *et al.* 2000), including *VEL1*, encoding an uncharacterized cell-surface protein, its paralog *YOR387C*, and the genes encoding the alcohol dehydrogenase Adh4, the putative GPI-anchored protein Zps1, and the zinc transporter Zrt1. Other genes included *YLR194C* , *PUN1*, *TIR1* and *DAN1* encoding cell wall proteins, and *ANB1* encoding a translation elongation factor. Finally, three uncharacterized genes, *YBR056W-A*, *YNL018C*, and *YOL159C*, were also repressed by both perturbations. The nonreference gene 59A_0034g, which possess a Flo11-like domain (pfam10182) and was strongly repressed in the two disomes, also behaved like a member of this group.

The behavior of the phosphate-regulated genes (*PHO84*, *PHO89*, *SPL2*, *GIT1*, *PHO5*, and *VTC1*) is particularly interesting. These genes are repressed both by *DIG1* overexpression and also by the chromosome XVI disome with the hemizygous deletion, both relative to F45, *i.e.*, their expression is repressed by increased copy number of both *DIG1* and one or more other genes on chromosome XVI. However, the full chromosome XVI disome, in which the copy number of both *DIG1* and the other regulatory gene(s) is increased, shows less, rather than more, repression of these genes, relative to F45 than is seen in the disome with the hemizygous *DIG1* deletion. Therefore, the expression of these phosphate genes appears to reflect a nonlinear interaction between *DIG1* copy number and that of one or more other genes on chromosome XVI.

### Characterization of genes commonly differentially expressed in the chromosome XV and XVI disomes

**Figure 5:**
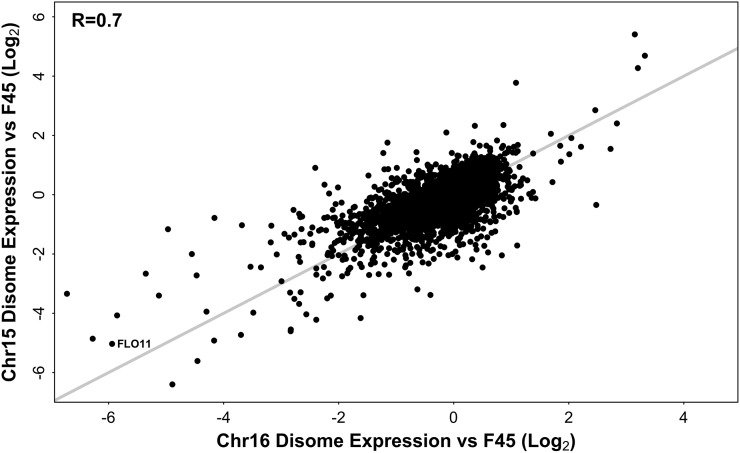
Differential gene expression in the chromosome XV and chromosome XVI disomes, relative to F45. Genes on disomic chromosomes had expression ratios adjusted downward twofold. *x* = *y* line shown in gray.

In our model of the mechanisms by which aneuploidy can affect gene expression (Figure 1B), we specified two mechanisms that could produce effects on gene expression unique to each aneuploid and two mechanisms that could produce similar effects on transcription across multiple aneuploids. To assess the relative contribution of shared *vs.* disome-specific factors in the gene expression patterns of our disomes, we examined the degree of similarity between the expression patterns of the two disomes. After accounting for direct copy number effects, by twofold downward adjustment of expression from the disomic chromosomes, the differential expression profiles of the two disomes, relative to F45, were similar (Figure 5), with an R of 0.70 (Pearson test, two-tailed, t = 74.5175, df = 5776, p-value < 1e−15). The same relationship (*R* = 0.71) was seen when only genes on chromosomes I–XIV were considered (Figure S1). More genes were repressed in each disome (ChrXV: 2171, ChrXVI: 1852) than induced (ChrXV: 1307, ChrXVI: 1149), at the multiple hypothesis corrected p < 0.01 level. Given their similarity, it appears that the differential gene expression patterns of the two disomes are dominated by shared, rather than disome-specific, factors.

To begin to understand the factors contributing to the shared differential expression profiles of the two disomes, we characterized the genes commonly induced and repressed in those strains. After downward adjustment of expression from chromosomes XV and XVI in the disomes, a total of 210 genes showed a >twofold decrease in expression in both strains relative to F45, statistically significant at the (multiple hypothesis corrected) p < 0.01 level (Table S1). This set was enriched for several GO terms including GO:0006099 “tricarboxylic acid cycle” (p-value = 1.71e−05), GO:0019953 “sexual reproduction” (p-value = 8.63e−04), GO:0006081 “cellular aldehyde metabolic process” (p-value = 7.78e−05), and GO:0030312 “external encapsulating structure” (p-value = 5.62e−07). Several genes involved in phosphate (*GIT1*, *PHM6*, *PHM7*, *PHM8*, *PHO5*, *PHO8*, *PHO84*, *PHO89*, and *SPL2*) and thiamine (*THI4*, *THI5*, *THI7*, *THI11*, *THI12*, and *THI13*) metabolism were also significantly repressed in both disomes.

The set of genes downregulated in both disomes (Table S1) also contained several genes with known roles in the development of complex colony phenotypes. These included the *FLO10* and *FLO11* flocculin genes, believed to encode effectors of complex colony morphologies (Lo and Dranginis 1998; Guo *et al.* 2000; Granek and Magwene 2010) (Halme *et al.* 2004; Voordeckers *et al.* 2012). Similarly, the gene encoding the ammonium permease Mep2, which regulates pseudohyphal growth in response to ammonium limitation (Lorenz and Heitman 1998a), and the genes encoding the Tec1 and Mga1 transcription factors, known to be positive regulators of *FLO11* gene expression and complex colony phenotypes (Gavrias *et al.* 1996; Lorenz and Heitman 1998b; Rupp *et al.* 1999; Borneman *et al.* 2006), were consistently repressed in the disomes. Expression of *BSC1*, which encodes an uncharacterized protein with a Flo11-like domain (pfam10182), was also consistently reduced. In addition, one of the unmapped, nonreference genes that we identified (59A_0034g), which encodes a protein that also possesses the Flo11-like domain (pfam10182) (File S1 and File S2), was also strongly repressed in the two disomes. As the chromosomes on which this and the other unmapped nonreference genes reside are unknown, no copy number adjustments were made for their expression in the disomes.

Only 25 genes showed at least a twofold increase in expression in both disomic strains relative to F45, statistically significant at the (multiple hypothesis corrected) p < 0.01 level (Table S2). The only significantly enriched GO terms were GO:0005576 “extracellular region” (p-value = 1.41e−02), GO:0031225 “anchored component of membrane” (p-value = 4.18e−02), and GO:0009277 “fungal-type cell wall” (p-value = 1.23e−02). These 25 genes included *HPF1* and *HPF1*’ (*YIL169C*), encoding cell wall mannoproteins and one of the unmapped, nonreference genes that we identified (EC1118_1O30_0001g), predicted to encode a protein with strong sequence similarity to Hpf1 (File S1 and File S2). It is interesting that cell surface mannoproteins were found both among the set of genes most highly repressed in the two disomes (*e.g.*, *FLO11* and *FLO10*) and among the genes most strongly induced (*e.g.*, *HPF1* and *HPF1*’)

### Relative strength of karyotype *vs.* genotype and environment on gene expression

The strong similarity between the transcriptional profiles of our two disomes raises questions about how interactions between genotype, environment, and ploidy affect gene expression. One possible explanation for the similar expression profiles of our disomes is that increasing the copy number of a whole chromosome specifies a strong transcriptional response that is relatively independent of genotype and environment. If this is true, we would expect each of our disomes to have a similar transcriptional profile in other studies, and the similarity between the two disomes to be maintained in those studies. To test this hypothesis, we compared our data to the differential transcriptional profiles of chromosome XV and chromosome XVI disomes in a previous study that used a different genetic background (W303) and a different environmental condition (batch growth) (Torres *et al.* 2007). Similar to our results (Figure 5), the differential expression profiles of the two disomes in that study were also similar (positively correlated with an *R* of 0.57) (Torres *et al.* 2007). However, we observed no correlation between the transcriptional profiles of the chromosome XV disomes (*R* = 0.011) or between the transcriptional profiles of the chromosome XVI disomes (*R* = 0.00038) across the two studies. These results suggest that the expression changes that accompany different aneuploid states are highly dependent on genotype and environment, but may be consistently similar to each other within any specific combination of genotype and environment.

### Contribution of colony morphology and cell environment to the differential expression profiles of the disomes

One of the mechanisms by which aneuploidy could affect gene expression patterns is indirectly through its effects on phenotype (Figure 1B). Both of the disomic strains in our study form smooth colonies, while the F45 euploid forms fluffy colonies. The similar transcriptional profiles of the disomes could therefore be a result of their shared colony morphology, perhaps reflecting a transcriptional response to the smooth *vs.* fluffy cellular environment. To test this idea, we examined gene expression in smooth colonies resulting from deletions of downstream effectors of the fluffy colony morphology. We reasoned that expression changes in these mutants, relative to F45, would give us the cleanest readout of any transcriptional response to the smooth colony “environment.”

To test this hypothesis, we constructed deletion mutants of *FLO11*, encoding a cell surface flocculin important for the formation of complex colony morphologies (Lo and Dranginis 1998; Granek and Magwene 2010; Voordeckers *et al.* 2012), and *CIS3*, encoding a mannose-containing glycoprotein and a constituent of the cell wall that we identified in a separate screen for mutations affecting colony morphology (Z. Tan and A. M. Dudley, unpublished results). Both of these deletions caused F45 colonies to lose their structured (fluffy) colony morphology, but retain their irregular boundaries (Figure 1A). As before, we performed RNA-seq on RNA isolated from these colonies after 4 d of growth on solid medium (YPD, 2% glucose).

**Figure 6:**
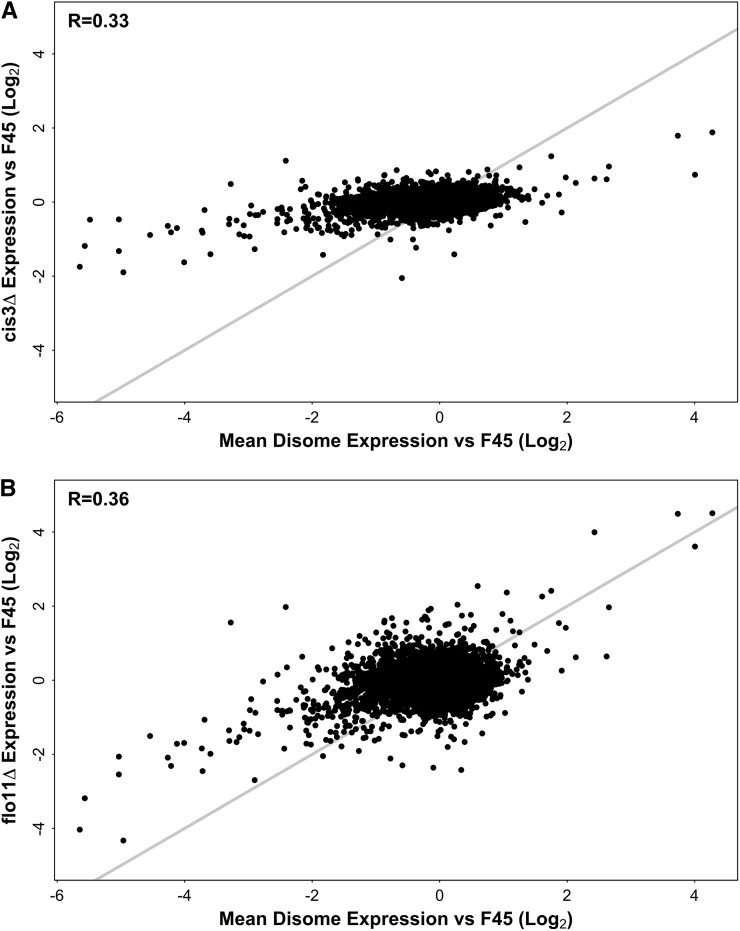
Differential gene expression in (A) *cis3*Δ and (B) *flo11*Δ colonies relative to F45, compared to the mean disome differential gene expression profile. *x* = *y* line shown in gray.

We first examined the effect of the *cis3*Δ. Despite the strong effect on colony morphology, little differential expression was seen in the *cis3*Δ strain, relative to F45. Only 117 genes showed a change in gene expression that was significant at the (multiple hypothesis corrected) p < 0.01 level, and only 10 of these involved more than a twofold change in expression (Table S3). To compare the effect of the *cis3*Δ to the shared expression pattern displayed by the disomes, we first calculated mean gene expression change relative to F45 for each gene across the two disomic strains. Expression from disomic chromosomes was first adjusted downward twofold to control for the direct effects of chromosome copy number. Much stronger gene expression change was seen in this common disome profile (mean absolute log_2_FC = 0.43) than in the *cis3*Δ (mean absolute log_2_FC = 0.22). Similarly, while the common disome and *cis3*Δ differential expression profiles were statistically significantly correlated (Pearson test, two-tailed, : *t* = 26.4532, df = 5772, p-value < 1e−15), this correlation was modest (*R* = 0.33) (Figure 6A), leaving most of the variance in the common disomic expression profile unexplained. Taken together, these results suggest that the fluffy *vs.* smooth cell environment does not in itself induce substantial changes in gene expression and that little of the strong, shared differential expression profile of the two disomes can be explained by their shared (smooth) colony morphology.

### Evidence for a signaling role for Flo11 in colony development

Given the low level of differential gene expression in the *cis3*Δ and the similar morphologies of the *flo11*Δ and *cis3*Δ colonies, we were surprised to observe a much stronger differential expression pattern in the *flo11*Δ (mean absolute log_2_FC of 0.38 *vs.* 0.22). A total of 70 genes were significantly (multiple hypothesis corrected p < 0.01) and strongly (> twofold) induced in the *flo11*Δ colonies, relative to F45, while 109 genes were significantly and strongly downregulated (Table S4). Comparing the differential expression pattern of the *flo11*Δ to that of the *cis3*Δ revealed a modest (*R* = 0.42), but significant correlation (Pearson test, two-tailed, : *t* = 35.2996, df = 5772, p-value < 1e−15) (Figure S2). This weak correlation, and the much greater degree of differential gene expression seen in the *flo11*Δ strain, strongly suggest that the differential expression profile of the *flo11*Δ strain does not simply reflect a response to the smooth colony environment that is shared with the *cis3*Δ colonies.

Similarly, comparing the differential expression pattern in the *flo11*Δ strain to the mean disome profile also revealed a modest (*R* = 0.36) but significant correlation (Pearson test, two-tailed, : *t* = 29.687, df = 5772, p-value < 1e−15). However, most of the genes whose transcription was most strongly changed in the two disomes, relative to F45, were also strongly differentially expressed, and in the same direction, in the *flo11*Δ colonies (Figure 6B). This included the *FLO10* and *YLR042C* cell wall genes and the *TEC1* and *MGA1* regulators of colony morphology. These results demonstrate that, like the disomes, deletion of *FLO11* affects the expression of genes involved in complex colony morphology development (*e.g.*, *FLO10*) and the signaling pathways regulating morphology development (*e.g.*, the “filamentous growth” MAPK cascade that includes Tec1 (Madhani and Fink 1997). As Flo11 is a downstream, cell surface effector of these pathways, this suggests that feedback mechanisms are operating, so that, in the *flo11*Δ strain, the signaling pathways regulating fluffy colony development themselves appear to be set to specify the smooth state, with downregulation of positive regulators such as *TEC1* and of mechanistic genes such as *FLO10*. These feedback mechanisms might be responding to some signal common to the disomes and the *flo11*Δ strain, perhaps even to *FLO11* expression level itself, which is strongly repressed in both disomes (Figure 5). Notably, in a subset of Flo11 molecules, the extracellular portion of the protein is shed from the cell surface into the surrounding medium, rather than remaining intact on the cell surface (Karunanithi *et al.* 2010). Msb2 and Hsk1, which (like Flo11) are cell surface glycoproteins, also shed their extracellular domains in this way and are known to have roles in MAPK signaling (Vadaie *et al.* 2008; Pitoniak *et al.* 2009).

Interestingly, the expression of *CIS3* is not significantly affected by the *FLO11* deletion, indicating that high *CIS3* expression, while necessary for fluffy colony formation, can also occur in smooth strains, *i.e.*, high *CIS3* expression is not sufficient for fluffy colony formation. In the *CIS3* deletion strain, *FLO11* expression is significantly, but only weakly, reduced (to 72% of the F45 level). This suggests either that high *FLO11* expression is also necessary, but not sufficient, for fluffy colony formation, or that even a very small reduction in *FLO11* expression level in F45, such as that seen in the *cis3*Δ strain, is sufficient to cause the fluffy-smooth transition.

### Differential expression of ESR genes in the disomes

**Figure 7:**
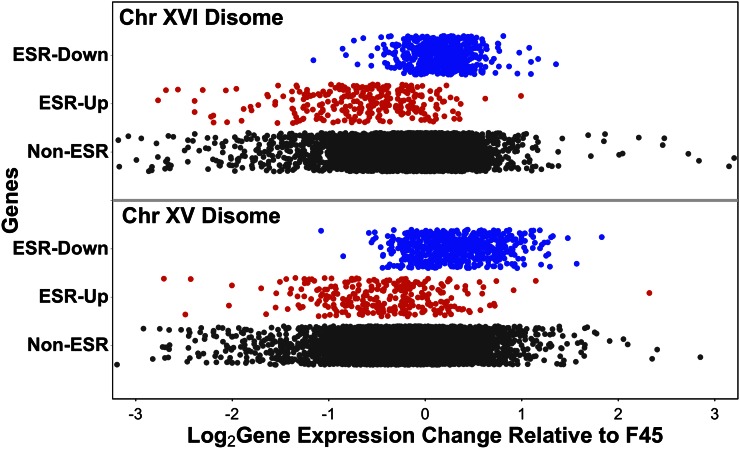
Differential expression of environmental stress response (ESR) genes in the chromosome XV and chromosome XVI disomes, relative to F45. Genes induced in ESR in red, genes repressed in blue, other genes in black. Genes on disomic chromosomes had expression ratios adjusted downward twofold. Chr, chromosome.

In our initial model (Figure 1B), the final mechanism that we proposed to mediate the effects of aneuploidy on gene expression is through nonspecific responses to aneuploidy *per se*. Previous studies have suggested that aneuploidy in yeast triggers transcriptional effects similar to the environmental stress response (ESR) (Gasch *et al.* 2000) and that this response is not specific to disomy of particular chromosomes, but instead is a generalized response to the noneuploid state (Torres *et al.* 2007). This mechanism could, potentially, explain the common differential gene expression pattern seen in our two disomes. To test this hypothesis, we examined expression of the 868 ESR genes in both of our disomic strains. After adjusting expression of genes on disomic chromosomes downward twofold, we observed a significant difference in expression between the set of genes that are downregulated in the ESR and non-ESR genes (ChrXV disome: Mann–Whitney test, two-tailed, W = 2070000, p-value < 1e−15; ChrXVI disome: Mann–Whitney, two-tailed, W = 1943400, p-value < 1e−15). We also observed a significant difference in expression between the set of genes that are upregulated in the ESR *vs.* non-ESR genes (ChrXV disome: Mann–Whitney test, two-tailed, W = 477460, p-value < 1e−15; ChrXVI disome: Mann–Whitney test, two-tailed, W = 314290, p-value < 1e−15). Surprisingly, however, the direction of effect was opposite to that expected, *i.e.*, the genes downregulated in the ESR were induced in the disomes and the genes upregulated in the ESR were repressed in the disomes (Figure 7). Therefore, it appears that the ESR is induced in euploid F45, relative to the two disomes, rather than vice versa.

Interestingly, this reversal of effect has previously been observed for a subset of the ESR genes. In the study that identified induction of the ESR response in aneuploid W303 strains grown in batch culture, a second generalized response to aneuploidy was observed when the same strains were grown in phosphate-limited chemostats (Torres *et al.* 2007). Interestingly, there was substantial overlap between the ESR and chemostat-response gene lists, but with a reversal of direction of effect. 98/176 of the genes upregulated in the W303 aneuploid chemostat response were genes downregulated in the ESR, while no genes were upregulated in both responses. Similarly, 95/222 of the genes downregulated in the W303 chemostat response were genes upregulated in the ESR, while only two genes were downregulated in both responses. Therefore, it appears that genes can be consistently upregulated across multiple aneuploids in one environmental condition (batch growth) while being consistently downregulated across the same aneuploids in a different environmental condition (chemostats).

In our disomes, we observed significant repression of the 222 genes downregulated among aneuploids in the chemostat study (ChrXV disome: Mann–Whitney test, two-tailed, W = 440240, p-value = 7.2e−08; ChrXVI disome: Mann–Whitney test, two-tailed, W = 292544, p-value < 1e−15) and significant induction of the 176 genes upregulated in that study (ChrXV disome: Mann–Whitney test, two-tailed, W = 597023, p-value = 3.9e−10; ChrXVI disome: Mann–Whitney test, two-tailed, W = 516041, p-value = 0.018). Therefore, it appears that our samples, from aneuploid colonies, may share a transcriptional response to aneuploidy with chemostat-grown W303 strains, but not with the same strains grown in batch culture.

### Colony morphology and the ESR

The similar effects on ESR gene expression seen in the two disomes could reflect the shared smooth colony morphology of the disomes, compared to the fluffy morphology of F45. To test this possibility, we examined the effect of the *CIS3* deletion on expression of the ESR genes, as *cis3*Δ colonies are also smooth and appear to represent our cleanest readout of the effect of the smooth environmental state. However, in the *cis3*Δ mutant, little change in expression of the ESR genes was observed relative to F45 (Figure S3), suggesting that colony morphology is not substantially driving the effect of the disomes on ESR gene expression.

### Role of DIG1 in differential expression of ESR genes in the chromosome XVI disome

**Figure 8:**
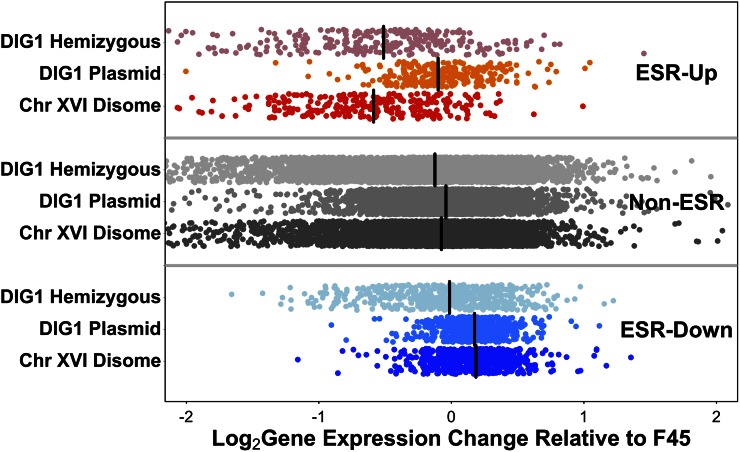
Differential expression, relative to F45, of environmental stress response (ESR) genes in the chromosome XVI disome, the disome hemizygous for *DIG1* (*DIG1/dig1*Δ), and in the *DIG1* overexpression strain. Genes induced in ESR in red, genes repressed in blue. Genes on disomic chromosomes had expression ratios adjusted downward twofold. Median values shown by black bars. Chr, chromosome.

To further explore the mechanisms underlying the effect of our two disomes on ESR gene expression, we examined the importance of *DIG1* copy number to the effect of the chromosome XVI disome. We observed that overexpression of *DIG1* from a plasmid led to induction of the ESR-repressed genes, and to a level similar to that seen in the full chromosome XVI disome. However, the ESR-induced genes, which are repressed in the disome, show little repression after *DIG1* overexpression (Figure 8). Interestingly, the reciprocal pattern was seen with the hemizygous deletion of *DIG1*, where there are two copies of chromosome XVI but only one copy of *DIG1*. In this strain, relative to F45, ESR-induced genes were repressed, as in the chromosome XVI disome; however, in contrast to the full disome, ESR-repressed genes showed little induction (Figure 8). Therefore, it appears that the effect of the chromosome XVI disome on differential expression of the ESR genes can largely be split into two separate copy number mechanisms, with induction of the ESR-repressed genes caused by increasing the copy number of *DIG1*, and repression of the ESR-induced genes caused by increasing the copy number of a gene or genes found elsewhere on chromosome XVI.

## Discussion

Aneuploidy, such as that underlying Down syndrome or found in most tumor cells, perturbs the expression of large numbers of genes simultaneously. In this study, we used a yeast model to dissect distinct components contributing to the impact of two different aneuploidies on gene expression. Our analysis was able to isolate effects on transcription that were shared between the two aneuploidies and effects unique to each. Additionally, we identified nonlinear interactions between the copy number of a transcription factor and the copy number of the other genes on the same chromosome, an interaction affecting the expression of multiple genes.

One of the factors we expected to be unique to each disome was the direct effect of increased copy number on expression of genes from the affected chromosome. As expected, expression of genes on the disomic chromosomes was elevated relative to the rest of the genome. Previous work has indicated that, in yeast, average gene expression levels from a chromosome correlate closely with chromosome copy number, when that copy number is perturbed by aneuploidy (Hughes *et al.* 2000; Torres *et al.* 2007, 2016; Rancati *et al.* 2008; Dephoure *et al.* 2014). However, a recent study argued that widespread dosage compensation occurs in some yeast strains, so that certain genes, particularly those that might be harmful if overexpressed, show lower than expected increases in expression when their copy number increases through aneuploidy (Hose *et al.* 2015; Gasch *et al.* 2016). However, in that same study, the expression levels of a similar number of genes increased more than their copy number. An alternative explanation for these results is that when the copy number of a chromosome increases, expression of genes on that chromosome is increased proportionally; however, some genes may be additionally affected (up- or downregulated) by other effects of the aneuploidy (Torres *et al.* 2016), such as changes in the copy number of genes encoding transcriptional regulators (Rancati *et al.* 2008).

In our study, we observed median increases in gene expression levels from the disomic chromosomes, relative to F45, somewhat lower than the expected twofold. However, when expression was compared between the two disomes, median expression changes closer to twofold were observed and the SD of the log ratios was reduced. This is consistent with expression of genes on the disomic chromosomes being affected by two phenomena. First, the effect of doubling gene copy number appears to increase gene expression twofold. Second, genes on chromosomes XV and XVI, just as genes elsewhere on the genome (Figure 5), show similar changes in gene expression in both disomes and this effect is independent of chromosome copy number. When this second effect is taken into account, by comparing gene expression between the disomes, the direct effect of copy number is seen more clearly.

We also expected to find additional transcriptional effects specific to each disome, caused by perturbing the copy number of transcriptional regulatory genes present on each of the two disomic chromosomes. To explore the role of regulatory gene copy number in gene expression changes caused by aneuploidy, we varied the copy number of the *DIG1* transcriptional regulator in the context of an F45 background that was euploid or disomic for chromosome XVI (on which *DIG1* is located). A significant correlation was seen between the perturbations in the two backgrounds with magnitudes that were consistent with the relative changes in *DIG1* expression level. However, the strength of the correlation was modest, suggesting that the effect of *DIG1* copy number changes might be highly dependent on the copy number of genes on the rest of chromosome XVI or that the effects of changes in *DIG1* copy number are nonlinear. We specifically identified several genes whose expression was consistent with a nonlinear interaction between *DIG1* copy number and the copy number of chromosome XVI. This included several genes in the phosphate regulon, suggesting interactions between *DIG1* copy number and the copy number of a regulatory gene or genes elsewhere on chromosome XVI specifically involved in phosphate gene expression.

In addition to the transcriptional effects unique to each disome, we also expected to see effects in common between the disomes, and in fact the differential transcriptional profiles of our two disomes were similar. One potential source for these common transcriptional effects is the ESR, in which the expression of a large number of transcripts changes in response to various stress conditions (Gasch *et al.* 2000). A previous publication identified induction of the ESR in multiple, distinct aneuploid derivatives of the laboratory yeast strain W303 grown in liquid batch culture (Torres *et al.* 2007). Induction of this response appears to reflect the relatively slow growth rates of these strains, relative to the euploid control. Previous studies in yeast have shown that slow growth can induce the ESR (Regenberg *et al.* 2006), and analogous stress responses and impaired proliferation have also been seen in response to aneuploidy in plant and mammalian cells (Sheltzer *et al.* 2012). As such, a growth impairment leading to the induction of a stress response might be regarded as a conserved nonspecific response to aneuploidy.

In contrast to the results seen in the W303 laboratory strain background, studies using a number of aneuploid wild strains of yeast grown in batch culture failed to identify induction of the ESR (Hose *et al.* 2015). Notably, these strains also did not appear to suffer significant growth impairment associated with their aneuploidy. Similarly, when the W303 strains were grown in phosphate-limited chemostats, eliminating the growth disadvantage of the aneuploid strains, induction of the ESR was not observed (Torres *et al.* 2007). However, in a study assaying plate growth of S288c-derived aneuploid yeast strains (Pavelka *et al.* 2010), differential expression of ESR genes was also not consistently observed, including among aneuploid strains with strong growth defects. Taken together, and as argued by Pavelka *et al.* (2010), it appears that induction of the ESR is not an obligate response to aneuploidy. Instead, the induction of the ESR by aneuploidy in some studies is likely to be a consequence of specific combinations of genotype and environment.

In our study, using colonies grown on solid medium, we did observe differential expression of the ESR genes between the euploid and aneuploid strains. Surprisingly, however, the direction of effect was reversed from that expected, with genes upregulated in the ESR having lower expression in our aneuploid strains and genes downregulated in the ESR having higher expression. This effect (the reversal of the ESR) is not easily explained by improved growth of the aneuploids, as we have previously shown that the chromosome XVI disome is at a growth disadvantage in plate growth, compared to euploid F45 (Tan *et al.* 2013). This implies that, in our genetic background, aneuploidy, the colony morphology state, or a combination of both might relieve a stress condition experienced by the F45 euploid strain and possibly overwhelm any opposing impact on the ESR of the growth defect associated with the disomy. Interestingly, while growth of aneuploid W303 strains in phosphate-limited chemostats did not induce the ESR, a separate transcriptional response was seen across multiple aneuploids (Torres *et al.* 2007). Fifty percent of the genes in this response were ESR genes but, as was the case in our study, the direction of effect was reversed. Finally, our results suggest that the effect of the chromosome XVI disome on the ESR response can be split into ESR-repressed genes, which appear to respond to the effect of *DIG1* copy number, and ESR-induced genes, which appear to respond to the copy number of a gene or genes elsewhere on chromosome XVI. This observation argues against the ESR-response in our strains as a true generalized response to the euploid/aneuploid switch.

Finally, comparison of our results with those of a previous study demonstrated that the expression changes that accompany specific aneuploid states are highly dependent on genotype and environment. In turn, this has implications for the relationship between aneuploidy and cancer as it suggests that the effect of any given aneuploidy might be highly genotype- and tissue type-specific. Therefore, as a prognostic tool or for driving cancer treatment decisions, knowledge of ploidy may be relatively uninformative when viewed in isolation from those other factors.

## Supplementary Material

Supplemental material is available online at www.g3journal.org/lookup/suppl/doi:10.1534/g3.116.036160/-/DC1.
