# Supplementary figures and images for: Dissecting Gene Expression Changes Accompanying a Ploidy-Based Phenotypic Switch
Source: G3 (Bethesda). 2016 Nov 11;7(1):233–46. doi: 10.1534/g3.116.036160 (PMC5217112; doi:10.1534/g3.116.036160)

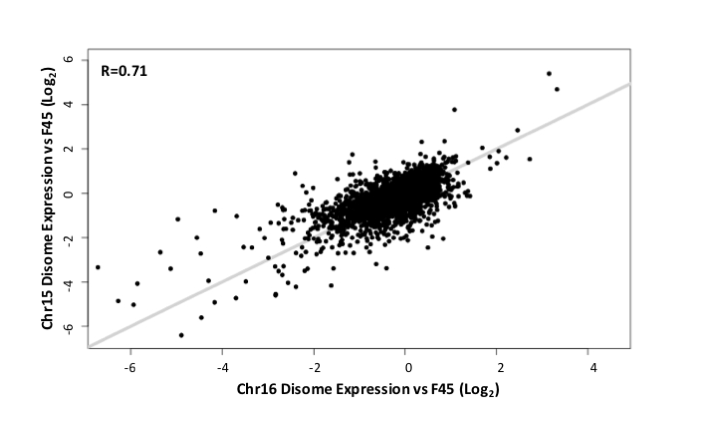

Supplement: Supplementary file 1 [file 233FigureS1.tiff]

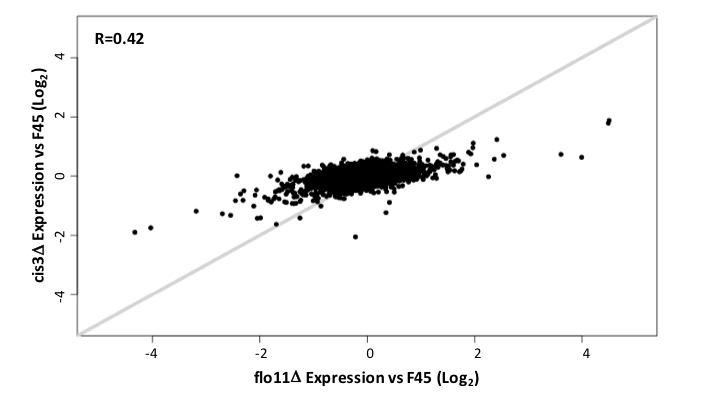

Supplement: Supplementary file 2 [file 233FigureS2.tiff]

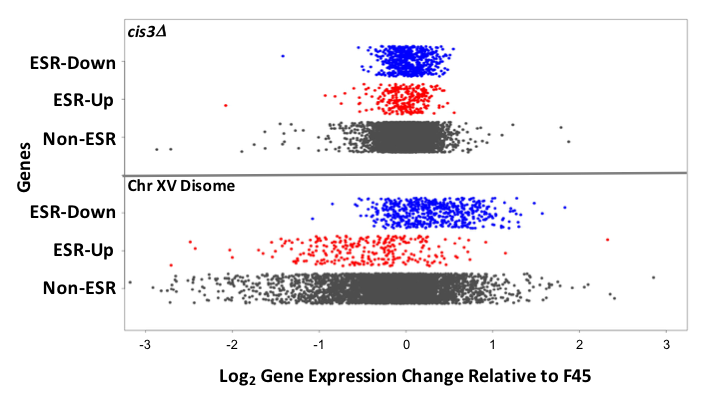

Supplement: Supplementary file 3 [file 233FigureS3.tiff]
