# Supplementary material for: Dissecting Gene Expression Changes Accompanying a Ploidy-Based Phenotypic Switch
Source: G3 (Bethesda). 2016 Nov 11;7(1):233–46. doi: 10.1534/g3.116.036160 (PMC5217112; doi:10.1534/g3.116.036160)
Supplement: Supplementary file 4 [file 233FileS1.docx]

File S1. FASTA genome sequence, extended to include non-reference contigs as pseudo-chromosomes. (.zip, 3.7 MB)

<http://www.g3journal.org/lookup/suppl/doi:10.1534/g3.116.036160/-/DC1/FileS1.zip>
