# Supplementary material for: Dissecting Gene Expression Changes Accompanying a Ploidy-Based Phenotypic Switch
Source: G3 (Bethesda). 2016 Nov 11;7(1):233–46. doi: 10.1534/g3.116.036160 (PMC5217112; doi:10.1534/g3.116.036160)
Supplement: Supplementary file 6 [file 233FileS2.docx]

File S2. GFF file for genome sequence. (.zip, 1.63 MB)

<http://www.g3journal.org/lookup/suppl/doi:10.1534/g3.116.036160/-/DC1/FileS2.zip>
