# Supplementary material for: Dissecting Gene Expression Changes Accompanying a Ploidy-Based Phenotypic Switch
Source: G3 (Bethesda). 2016 Nov 11;7(1):233–46. doi: 10.1534/g3.116.036160 (PMC5217112; doi:10.1534/g3.116.036160)
Supplement: Supplementary file 10 [file 233TableS2.docx]

Table S2. Genes showing >2 fold increase in expression in each of the two disomes, relative to F45 with p-value of <0.01 in both comparisons. (.xlsx, 13 KB)

<http://www.g3journal.org/lookup/suppl/doi:10.1534/g3.116.036160/-/DC1/TableS2.xlsx>
